# The effect of hydroalcoholic extract and essential oil of *Heracleum persicum* on lipid profile in cholesterol-fed rabbits

**Published:** 2014

**Authors:** Valiollah Hajhashemi, Gholamreza Dashti, Salabali Saberi, Parvin Malekjamshidi

**Affiliations:** 1 *Department of Pharmacology, School of Pharmacy and Pharmaceutical Sciences, Isfahan University of Medical Sciences, Isfahan, I. R. Iran*; 2 *Department of Anatomy, School of Medicine, Isfahan University of Medical Sciences, Isfahan, I. R. Iran*

**Keywords:** *Essential oil*, *Heracleum persicum*, *Hyperlipidemia*

## Abstract

**Objective:** This study was designed to investigate the effect of hydroalcoholic extract and essential oil of *Heracleum persicum* (Apiaceae) on lipid profile of male hyperlipidemic rabbits.

**Materials and Methods:** Thirty rabbits were randomly divided into six groups of five each. One group received normal diet and the other groups fed with a high cholesterol (2% W/W) diet for 7 weeks. Vehicle, hydroalcoholic extract (500 and 1000 mg/kg), essential oil (200  l/kg), and lovastatin (5 mg/kg) were administered orally to animals and their effects on lipid profile were evaluated.

**Results:** Essential oil of *H. perscum* significantly (p<0.05) lowered serum triglyceride level and increased HDL-cholesterol concentration. Moreover, hydroalcoholic extract (1000 mg/kg), essential oil (200  l/kg), and lovastatin significantly (p<0.01) reduced serum concentration of total cholesterol and LDL-cholesterol.

**Conclusion:** These findings suggest that essential oil of the plant fruits may have some benefits in reducing cardiovascular risk factors.

## Introduction

Atherosclerosis has a key role in the pathogenesis of myocardial and cerebral infarction. There are several risk factors for development of atherosclerosis including age, male gender, hypertension, smoking, and hyperlipidemia (Brunton et al., 2011 ▶). It has been reported that high blood levels of triglyceride and cholesterol contribute to atherosclerotic changes of arteries (Cambien et al., 1986 ▶; Austin, 1989 ▶). Hypercholesterolemia, or more speciﬁcally elevated plasma low density lipoprotein cholesterol (LDL-C) and its oxidized form (ox-LDL) are important risk factors for the development and progression of atherosclerosis (Keys, 1997 ▶). It has been reported that two key initial events within the arterial wall during early atherogenesis are the recruitment and differentiation of circulating monocytes and the uptake of cholesterol and ox-LDL by tissue macrophages to form lipid-foam cells, involved in atheroma plaque generation (Napoli et al., 1997 ▶; Steinberg, 1997 ▶; Napoli, 1999 ▶). 

Therefore, one possible method of preventing atherosclerotic diseases would be administration of antioxidative substances and thereby making LDL less sensitive to oxidation. Moreover, chemical hypolipidemic drugs such as resins, statins, and ﬁbrates which reduce serum triglycerides and LDL-cholesterol to various extents and increase high-density lipoprotein (HDL)-cholesterol levels have an important role in prevention of atherosclerosis (Brunton et al., 2011 ▶). However, these drugs have numerous and signiﬁcant side effects. Efforts have been done to find drugs with a better safety profile and to achieve this goal, some investigators have switched their studies toward medicinal herbs. Several studies have shown that medicinal herbs and nutrients including artichoke (Thompson Coon, 2003 ▶), fenugreek (Basch et al., 2003 ▶), garlic (Orekhov and Grunwald, 1997 ▶), ginger (Bhandari et al., 1998 ▶), dillweed oil (Hajhashemi and Abbasi, 2008 ▶), caraway (Lemhadri et al., 2006 ▶), coriander (Chithra and Leelamma, 1997 ▶), cumin (Dhandapani et al., 2002 ▶), and soy bean (Anderson et al., 1995 ▶) have beneficial effects on serum lipid levels and preventive effect against atherosclerosis. 

Several of above medicinal herbs including dill, caraway, coriander, and cumin belong to Apiaceae family. Moreover, recently it was reported that *Prangos ferulacea*, another plant of Apiaceae family showed improvement of serum glucose and lipids in alloxan-induced diabetic rats. *H. persicum* is a plant of Apiaceae family and it has been the subject of many investigations. 

Heracleum persicum Desf. ex Fischer is a perennial flowering plant native to Iran. The height of the plant reaches 150-200 cm. This large white-flowered plant grows wild in humid mountainous regions. Its flowering time is July to September. Fruits are broadly obovate, 7 to 8 mm long and 2-parted with slightly ridged schizocarp (Asgarpanah et al., 2012 ▶). Fruits are widely used as spices and the young stems are also used for making pickles. In Iranian folk medicine, the fruits of *H. persicum* were used as a carminative and pain killer herbal drug (Naraghi, 1972 ▶; Zargari, 1988 ▶). Chemical composition of different parts of *H. persicum* has been investigated by several authors. Some reports indicate the presence of six furanocoumarins and flavonoids in the fruits of *H. persicum* (Ghodsi, 1976 ▶; Merijanian et al., 1980 ▶). Essential oil is one of the most important constituents of the fruits, leaves, flowers, and roots of *H. persicum* (Scheffer et al., 1984 ▶; Mojab et al., 2002 ▶, 2003; Sefidkon et al., 2004 ▶).

Antioxidant activity of some furanocoumarins isolated from *H. persicum* has been reported by Souri et al. (2004) ▶. Moreover, it has been shown that furanocoumarins have protective effect against lipid peroxidation (Vimal and Devaki, 2004 ▶; Phuwapraisirisan et al., 2006 ▶). Based on antioxidant activity of *H. persicum* and considering the presence of several furanocoumarin compounds in its fruits and also lipid lowering activity of above-mentioned plants of Apiaceae family, this study was aimed to evaluate hypolipidemic properties of hydroalcoholic extract and essential oil of *H. persicum* fruits in rabbits.

## Materials and Methods

**Plant material and preparation of extract and essential oil**

Fruits of *H. persicum* were collected from north of Tehran. The plant identity was confirmed by the Botany Department of Isfahan University. A voucher specimen (No. 1703) was deposited in the herbarium of Faculty of Pharmacy and Pharmaceutical Sciences, Isfahan University of Medical Sciences, Isfahan, Iran for further reference.

For preparation of hydroalcoholic extract, air-dried and powdered fruits of the plant (200 g) were macerated with 1500 ml of EtOH-H_2_O (7:3) for 48 hours. The extract was then shacked, filtered, and evaporated in a rotating evaporator under reduced pressure until dryness (Sajjadi et al., 1998 ▶). Evaporation and solvent removal of hydroalcoholic extract gave semi-solid masses (yield 7.5%). 

The essential oil was isolated by hydro distillation of the powdered fruits of the plant for 3 hours according to the method recommended in European Pharmacopoeia (2002). The yield value of the essential oil was 3.8% (v/w). The essential oil was kept in a cool and dry place and the hydroalcoholic extract was kept in a refrigerator.

**Animals**

Thirty male New Zealand white rabbits with a body weight of 1.8-2.2 kg were used in this study. Animals were purchased from Pasteur Institute (Tehran, Iran). Two animals were kept together in each large cage with daylight illumination and free access to water *ad libitum*. The temperature in the animal room was 22±2 ^o^C and the general health and activity of rabbits were monitored closely. All experimental procedures were conducted in accordance with the local guidelines of the animal ethical committee for animal experimentation in Isfahan University of Medical Sciences (Isfahan, Iran).

**Experimental design**

 After 2 weeks of adaptation to the standard diet, blood samples were collected from the rabbits’ ear artery to measure basal serum lipid levels. The animals were randomly divided into six groups of five each and fed as follows: 

Group 1: Normal animals fed with normal diet, 

Group 2: Control group fed with high cholesterol diet (HCD) plus vehicle (2 ml/kg). 

Group 3: Rabbits fed with HCD plus *H. persicum* essential oil (200  l/kg).

Group 4: Rabbits fed with HCD plus *H. persicum *hydroalcoholic extract (500 mg/kg). 

Group 5: Rabbits fed with HCD plus *H. persicum *hydroalcoholic extract (1000 mg/kg), and Group 6: rabbits fed with HCD plus lovastatin (5 mg/kg).

HCD was prepared by adding 2% (w/w) cholesterol (Merck, Germany) to standard diet. 

Hydroalcoholic extract and lovastatin were suspended in 1% sodium carboxymethyl cellulose to a ﬁnal volume of 2 mL/kg of body weight. All drugs were administered orally once daily and the duration of treatment was 7 weeks for each animal. 

**Determination of serum lipids**

 Serum cholesterol, triglyceride, and HDL-C were assayed by colorimetric methods (Bucolo and David, 1973 ▶; Allain et al., 1974 ▶; Rifai et al., 1992 ▶) using available commercial kits (Zistshimi, Iran). LDL-cholesterol was calculated by the method of Varley (1991) ▶. 

**Statistical analysis**

The results are presented as mean±SEM and statistically analyzed by one way analysis of variance (ANOVA) followed by Duncan’s test.

## Results

As it is seen in Table 1, mean values of serum cholesterol concentration at zero weeks (baseline values) are not significantly different between groups. A high cholesterol diet produced an eight-fold increase in serum cholesterol concentration of the rabbits. Seven weeks treatment of rabbits with *H. persicum* essential oil at a dose of 200 l/kg and hydroalcoholic extract at a dose of 1000 mg/kg reduced serum cholesterol concentration by 23% and 29%, respectively in comparison with control group. Lovastatin at a dose of 5 mg/kg decreased cholesterol level by 44%. Only in essential oil-treated group, a minimal weight loss was observed that was not significant.

**Table 1:** Effect of hydroalcoholic extract and essential oil of *H. persicum* on serum cholesterol concentration

**Group **	**0 week (mg/dl)**	**7 weeks (mg/dl)**
**Normal**	74±1.9	76±9.6
**Control**	82±4.0	598±20.0*
**HPEO (200 l/kg)**	79±1.0	460±11.2*#
**HPHE (500 mg/kg)**	74±0.6	523±11.6*
**HPHE (1000 mg/kg)**	78±3.2	423±37.4*#
**Lovastatin (5 mg/kg)**	85±2.9	337±16.1*#

Data are expressed as mean±SEM of five rabbits per group.

* p<0.001 compared with normal group.

# p<0.01 compared with control (hyperlipidemic) group.

Effect of different treatments on serum triglyceride concentration is summarized in Table 2. Baseline values are not significantly different between groups. A high cholesterol diet produced about three fold increase in serum triglyceride concentration. Although all treatments including *H. persicum* essential oil and hydroalcoholic extract and lovastatin have decreased serum triglyceride concentration in comparison with control group, the change was statistically significant (p<0.05) only for essential oil group.

**Table 2:** Effect of hydroalcoholic extract and essential oil of *H. persicum* on serum triglyceride concentration

**Group **	**0 week (mg/dl)**	**7 weeks (mg/dl)**
**Normal**	75±5.8	74±4.5
**Control**	79±3.5	223±12.0*
**HPEO (200 l/kg)**	102±3.8	110±4.1#
**HPHE (500 mg/kg)**	78±3.4	174±7.3*
**HPHE (1000 mg/kg)**	86±5.7	199±11.9*
**Lovastatin (5 mg/kg)**	82±4.7	163±6.9*

Data are expressed as mean±SEM of five rabbits per group.

* p<0.001 compared with normal group.

# p<0.05 compared with control (hyperlipidemic) group.

Hypercholesterolemic rabbits which received *H. persicum* essential oil had a significantly (p<0.05) higher HDL-cholesterol concentration in comparison with control hypercholesterolemic animals that only received a high cholesterol diet. Therefore, essential oil produced 43% increase in HDL-cholesterol concentration (Table 3).

**Table 3:** Effect of hydroalcoholic extract and essential oil of *H. persicum* on serum HDL-cholesterol concentration

**Group **	**0 week (mg/dl)**	**7 weeks (mg/dl)**
**Normal**	23.6±0.6	26.8±1.4
**Control**	26.0±1.3	77.0±2.1*
**HPEO (200 l/kg)**	24.1±0.8	110.4±3.4*#
**HPHE (500 mg/kg)**	19.0±1.2	97.0±3.0*
**HPHE (1000 mg/kg)**	22.6±1.0	55.4±2.7*
**Lovastatin (5 mg/kg)**	28.5±1.4	88.5±4.1*

Data are expressed as mean±SEM of five rabbits per group.

* p<0.001 compared with normal group.

# p<0.05 compared with control (hyperlipidemic) group.

Table 4 shows the effect of different treatments on LDL-cholesterol concentration. Essential oil (200 l/kg), hydroalcoholic extract (1000 mg/kg), and lovastatin significantly (p<0.01) reduced serum LDL levels in hypercholesterolemic groups and the percent of reduction was 31.3%, 29.4%, and 54.7%, respectively.

**Table 4:** Effect of hydroalcoholic extract and essential oil of *H. persicum* on serum LDL-cholesterol concentration

**Group **	**0 week (mg/dl)**	**7 weeks (mg/dl)**
**Normal**	35.3±4.8	34.4±12.1
**Control**	40.2±7.1	476.8±42.5*
**HPEO (200 l/kg)**	34.2±8.1	327.6±33.8*#
**HPHE (500 mg/kg)**	39.4±3.1	399.2±25.9*
**HPHE (1000 mg/kg)**	38.0±4.9	336.8±39.6*#
**Lovastatin (5 mg/kg)**	40.1±1.4	215.9±18.2*#

* p<0.001 compare with normal group.

# p<0.01 compared with control (hyperlipidemic) group.

## Discussion

The results of the present study clearly showed that essential oil of *H.* *persicum* increases HDL-cholesterol and decrease LDL-cholesterol and therefore reduces atherogenic index which is an important predictive factor of atherosclerosis. The hydroalcoholic extract of the plant at a dose of 1000 mg/kg could also significantly reduce total cholesterol and LDL-C. *H. persicum* belongs to Apiaceae family and in agreement with our findings, antihyperlipidemic activity has been reported for some other members of this family including dill (Hajhashemi and Abbasi, 2008 ▶), caraway (Lemhadri et al., 2006 ▶), coriander (Chithra and Leelamma, 1997 ▶), and cumin (Dhandapani et al., 2002 ▶). 

It has been reported that essential oil of *H. persicum* is mainly composed of octyl acetate and hexyl butyrate (Scheffer et al., 1984 ▶), however, to the best of our knowledge, the lipid lowering effect of these constituents has not been studied and it is not clear if one or both of these components contribute to the changes of lipid profile observed in our research.

The mechanism of hypolipidemic activity of *H. Persicum* essential oil and hydroalcoholic extract in reducing LDL-C is not clear. They may reduce LDL-C by inhibiting intestinal absorption of cholesterol with a mechanism similar to ezetimibe (Sweeney and Johnson, 2007 ▶). Another possible locus for action of the essential oil or active constituents of hydroalcoholic extract is the rate-limiting enzyme, hydroxyl-methyl-glutaryl-Co A reductase (HMG-COA reductase). Inhibition of this enzyme by well-known drugs such as statins reduces hepatic cholesterol synthesis and upregulates hepatic LDL-C receptors to enhance LDL-C uptake and thereby results in a lowered serum level of LDL-C (Brunton et al., 2011 ▶). 

Another beneficial effect of *H. persicum* is its antioxidant potential. Souri et al. (2004) ▶ reported antioxidant activity for some furanocoumarins of *H. persicum*. It has been documented that oxidized LDL (modified LDL) in subendothelial space of coronary and cerebral arteries is atherogenic. Oxidation of LDL is a required step for LDL uptake by the scavenger receptors of macrophages and foam cell formation. Foam cells then form fatty streak and atherosclerotic plaque (Brunton et al., 2011 ▶). Therefore it seems that antioxidant activity of *H. persicum* in addition to its lipid lowering effect can help in preventing cardiovascular events. 

In conclusion, *H. persicum* has hypolipidemic and antiatherosclerotic potentials and since its wide uses as spice, it is a good candidate for further researches regarding cardiovascular problems.

## References

[B1] Allain CC, Poon LS, Chan CS, Richmond W, Fu PC (1974). Enzymatic determination of total serum cholesterol. Clin Chem.

[B2] Anderson JW, Johnstone BM, Cook-Newell ME (1995). Meta-analysis of the effects of soy protein intake on serum lipids. N Engl J Med.

[B3] Asgarpanah J, Dadashzadeh Mehrabani G, Ahmadi M, Ranjbar R, Safi-Aldin Ardebily M (2012). Chemistry, pharmacology and medicinal properties of Heracleum persicum Desf Ex Fischer: A review. J Med Plants Res.

[B4] Austin MA (1989). Plasma triglyceride as a risk factor for coronary heart disease The epidemiologic evidence and beyond. Am J Epidemiol.

[B5] Basch E, Ulbricht C, Kuo G, Szapary P, Smith M (2003). Therapeutic applications of fenugreek. Altern Med Rev.

[B6] Bhandari U, Sharma JN, Zafar R (1998). The protective action of ethanolic ginger (Zingiber officinale) extract in cholesterol fed rabbits. J Ethnopharmacol.

[B7] Brunton LL, Lazo JS, Parker KL (2006). Goodman & Gilman,s the pharmacological basis of therapeutics.

[B8] Bucolo G, David H (1973). Quantitative determination of serum triglycerides by the use of enzymes. Clin Chem.

[B9] Cambien F, Jacqueson A, Richard JL, Warnet JM, Ducimetiere P, Claude JR (1986). Is the level of serum triglyceride a significant predictor of coronary death in "normocholesterolemic" subjects? The Paris Prospective Study. Am J Epidemiol.

[B10] Chithra V, Leelamma S (1997). Hypolipidemic effect of coriander seeds (Coriandrum sativum): mechanism of action. Plant Foods Hum Nutr.

[B11] Dhandapani S, Subramanian VR, Rajagopal S, Namasivayam N (2002). Hypolipidemic effect of Cuminum cyminum L on alloxan-induced diabetic rats. Pharmacol Res.

[B12] (2002). European Pharmacopoeia.

[B13] Ghodsi B (1976). Flavonoids of three Heracleum species: H. Persicum L., H. sphondylium L. and H. montanum. Bull. Trav Soc Pharm Lyon.

[B14] Hajhashemi V, Abbasi N (2008). Hypolipidemic activity of Anethum graveolens in rats. Phytother Res.

[B15] Kafash Farkhad N, Farokhi F, Tukmacki A, Soltani Band Kh (2012). Hydro-alcoholic extract of the root of Prangos ferulacea (L.) Lindl can improve serum glucose and lipids in alloxan-induced diabetic rats. Avicenna J Phytomed (AJP).

[B16] Keys A (1997). Coronary heart disease in seven countries. Nutrition.

[B17] Lemhadri A, Hajji L, Michel JB, Eddouks M (2006). Cholesterol and triglycerides lowering activities of caraway fruits in normal and streptozotocin diabetic rats. J Ethnopharmacol.

[B18] Merijanian A, Colasurdo T, Samtak P, Ullrich J, Spagnuolo J (1980). The furanocomarins of Heracleum persicum L. Rev Latinoam Quim.

[B19] Mojab F, Nickavar B (2003). Composition of the Essential Oil of the Root of Heracleum persicum from Iran. Iran J Pharm Res.

[B20] Mojab F, Rustaiyan A, Jasbi AR (2002). Essential oil of Heracleum persicum Desf ex Fischer leaves. Daru.

[B21] Napoli C, D'Armiento FP, Mancini FP, Postiglione A, Witztum JL, Palumbo G (1997). Fatty streak formation occurs in human fetal aortas and is greatly enhanced by maternal hypercholesterolemia. Intimal accumulation of low density lipoprotein and its oxidation precede monocyte recruitment into early atherosclerotic lesions. J Clin Invest.

[B22] Napoli C, Glass CK, Witztum JL, Deutsch R, D'Armiento FP, Palinski W (1999). Influence of maternal hypercholesterolaemia during pregnancy on progression of early atherosclerotic lesions in childhood: Fate of Early Lesions in Children (FELIC) study. Lancet.

[B23] Naraghi M (1972). Medicinal flowers and plants.

[B24] Orekhov AN, Grunwald J (1997). Effects of garlic on atherosclerosis. Nutrition.

[B25] Phuwapraisirisan P, Surapinit S, Tip-Pyang S (2006). A novel furanocoumarin from Feroniella lucida exerts protective effect against lipid peroxidation. Phytother Res.

[B26] Rifai N, Warnick GR, McNamara JR, Belcher JD, Grinstead GF, Frantz ID Jr (1992). Measurement of low-density-lipoprotein cholesterol in serum: a status report. Clin Chem.

[B27] Sajjadi SE, Movahedian-Atar AM, Yektaian A (1998). Antihyperlipidemic effect of hydroalcoholic extract and polyphenolic fraction from Dracocephalum Kotschyi Boiss. Pharmaceutica Acta Helvetiae.

[B28] Scheffer JJ, Hiltunen R, Aynehchi Y, von Schantz M, Svendsen AB (1984). Composition of Essential Oil of Heracleum persicum Fruits. Planta Med.

[B29] Sefidkon F, Dabiri M, Mohammad N (2004). Analysis of the oil of Heracleum persicum L (leaves and flowers). J Essent Oil Res.

[B30] Souri F, Farsam H, Sarkheil P, Ebadi F (2004). Antioxidant activity of some furanocoumarins isolated from Heracleum persicum. Pharm Biol.

[B31] Steinberg D (1997). Low density lipoprotein oxidation and its pathobiological significance. J Biol Chem.

[B32] Sweeney ME, Johnson RR (2007). Ezetimibe: an update on the mechanism of action, pharmacokinetics and recent clinical trials. Expert Opin Drug Metab Toxicol.

[B33] Thompson Coon JS, Ernst E (2003). Herbs for serum cholesterol reduction: a systematic view. J Fam Pract.

[B34] Varley H (1991). Practical Clinical Biochemistry.

[B35] Vimal V, Devaki T (2004). Linear furanocoumarin protects rat myocardium against lipidperoxidation and membrane damage during experimental myocardial injury. Biomed Pharmacother.

[B36] Zargari A (1988). Medicinal plants.

